# Cerebro-Cerebellar Pathways for Verbal Working Memory

**DOI:** 10.3389/fnhum.2018.00530

**Published:** 2019-01-08

**Authors:** Monika Sobczak-Edmans, Yu-Chun Lo, Yung-Chin Hsu, Yu-Jen Chen, Fu Yu Kwok, Kai-Hsiang Chuang, Wen-Yih Isaac Tseng, S. H. Annabel Chen

**Affiliations:** ^1^Department of Psychology, De Montfort University, Leicester, United Kingdom; ^2^Graduate Institute of Neural Regenerative Medicine, College of Medical Science and Technology, Taipei Medical University, Taipei, Taiwan; ^3^Institute of Medical Device and Imaging, National Taiwan University College of Medicine, Taipei, Taiwan; ^4^Centre for Research in Child Development, National Institute of Education, Nanyang Technological University, Singapore, Singapore; ^5^The Queensland Brain Institute, The University of Queensland, Brisbane, QLD, Australia; ^6^The Centre for Advanced Imaging, The University of Queensland, Brisbane, QLD, Australia; ^7^Institute of Brain and Mind Sciences, National Taiwan University College of Medicine, Taipei, Taiwan; ^8^Department of Radiology, National Taiwan University College of Medicine, Taipei, Taiwan; ^9^Molecular Imaging Center, National Taiwan University, Taipei, Taiwan; ^10^Psychology, School of Social Sciences, Nanyang Technological University, Singapore, Singapore; ^11^Lee Kong Chian School of Medicine, Nanyang Technological University, Singapore, Singapore; ^12^Centre for Research and Development in Learning, Nanyang Technological University, Singapore, Singapore

**Keywords:** cerebro-cerebellar pathways, diffusion spectrum imaging, deterministic tractography, working memory, effective connectivity

## Abstract

The current study examined the structural and functional connectivity of the cerebro-cerebellar network of verbal working memory as proposed by Chen and Desmond (2005a). Diffusion spectrum imaging was employed to establish structural connectivity between cerebro-cerebellar regions co-activated during a verbal working memory task. The inferior frontal gyrus, inferior parietal lobule, pons, thalamus, superior cerebellum and inferior cerebellum were used as regions of interest to reconstruct and segment the contralateral white matter cerebro-cerebellar circuitry. The segmented pathways were examined further to establish the relationship between structural and effective connectivity as well as the relationship between structural connectivity and verbal working memory performance. No direct relationship between structural and effective connectivity was found but the results demonstrated that structural connectivity is indirectly related to effective connectivity as DCM models that resembled more closely with underlying white matter pathways had a higher degree of model inference confidence. Additionally, it was demonstrated that the structural connectivity of the ponto-cerebellar tract was associated with individual differences in response time for verbal working memory. The findings of the study contribute to further our understanding of the relationship between structural and functional connectivity and the impact of variability in verbal working memory performance.

## Introduction

Working memory is defined as a system or systems that are necessary for keeping things in mind while performing cognitive tasks such as comprehension or learning (Baddeley, 2010). One of the most influential working memory models proposed by Baddeley conceptualized working memory as a system consisting of the central executive that controls cognitive processes, the visuospatial sketchpad that stores and manipulates images and the phonological loop that stores (phonological store) and manipulates (articulatory control rehearsal system) verbal information (Baddeley, 1986). The Sternberg paradigm (Sternberg, 1966) is often used to examine this model. A typical Sternberg task includes three distinct phases – encoding, maintenance and retrieval. In the encoding phase, participants are briefly presented with verbal information (usually 2–6 letters). In the maintenance phase, the verbal information is kept in mind through subvocal rehearsal, and in the retrieval phase, the stored information is utilized for making a response.

Based on this working memory model from cognitive psychology and virus tracings from primate studies (Brodal, 1979; Schmahmann, 1996), a cerebro-cerebellar framework for working memory was proposed (Desmond et al., 1997). Functional neuroimaging studies (e.g., Chen and Desmond, 2005b; Ng et al., 2016) used Sternberg paradigm (Sternberg, 1966) to examine functional brain changes in these cerebro-cerebellar networks for verbal working memory which was verified with transcranial magnetic stimulation (Desmond et al., 2005). Identified changes in brain functional activity included the left inferior frontal gyrus (IFG) and the right superior cerebellar lobule VI/crusI (the fronto-cerebellar loop) as well as the left inferior parietal sulcus and the right inferior cerebellar lobule VIIb/VIIIa (the parieto-cerebellar loop). These two loops exhibited increased activation during different verbal working memory stages. The fronto-superior cerebellar was associated with articulatory rehearsal processing and the parieto-inferior cerebellar loop with the phonological storage (Chen and Desmond, 2005b; Ng et al., 2016). Within the cerebro-cerebellar framework proposed by Desmond et al. (2005), the emphasis was placed on the IFG which is located in the ventrolateral parts of prefrontal cortex (PFC) rather than the dorsolateral PFC due to its involvement in the articulatory loop. This is because the IFG is linked with the articulatory rehearsal of verbal information (Fiez et al., 1996; Chen and Desmond, 2005b), whereas the dorsolateral PFC (e.g., BA9/46) is associated with retrieval of verbal information (Marvel and Desmond, 2010b) and also with maintenance of non-verbal information and rehearsal of non-verbal information (e.g., Fiez et al., 1996; Marvel and Desmond, 2010b; Ng et al., 2016; Sobczak-Edmans et al., 2016), hence it’s involvement in rehearsal processes for verbal working memory is less specific than involvement of IFG.

The fronto-cerebellar and parieto-cerebellar loops, when considered within the multi-component working memory model by Baddeley and Hitch (1974), have been found to support phonological rehearsal and phonological storage, respectively through functional neuroimaging studies (Chen and Desmond, 2005b; Macher et al., 2014; Ng et al., 2016). Macher et al. (2014) demonstrated that there is a relationship between the activity level in the inferior cerebellum (lobule VIIb) and item recognition capacity during Sternberg memory task. This suggested that the greater the neural activity in the right inferior cerebellum, the higher the number of recognized items. This relationship was no longer observed after anodal tDCS was applied to the inferior cerebellum. The involvement of the superior cerebellum in verbal working memory was also further verified with brain stimulation studies which showed that transient lesions to the right superior cerebellum significantly increase response time in Sternberg task (Desmond et al., 2005; Ferrucci et al., 2008) but does not affect accuracy. In addition to increased activation in right superior and inferior cerebellum, as well as in left frontal and parietal regions, the increased activation in thalamus and pons was observed by Chen and Desmond (2005b) during verbal working memory. Interestingly, Chen and Desmond (2005b) noted that the increased activation in the pons and thalamus during the encoding phase was also seen during the retrieval phase. Ide and Li (2011) provided evidence that through the connectivity with the thalamus and frontal regions, the cerebellum mediates error-related processing, hence it is possible that the cerebello-thalamo-cortical pathway is involved in executive function and error-related control (Ide and Li, 2011).

During working memory, increased activations observed in the cerebellar, frontal and parietal regions as well as in the pons and the thalamus are interesting, especially that these areas are inter-linked anatomically. Animal studies that used postmortem and *in vivo* tracers to label synaptically connected neurons have provided evidence that the cerebro-cerebellar white matter system includes contralateral cortico-ponto-cerebellar pathways (CPC) (Brodal, 1979; Schmahmann, 1996) that project from cortical regions through the pons to the cerebellum and contralateral cerebello-thalamo-cortical pathways (CTC) that connect the cerebellum through the thalamus and red nucleus with the cerebral cortex (Kelly and Strick, 2003; Middleton and Strick, 1994, 2001; Schmahmann, 1996). Primates studies have indicated that projections from frontal regions enter the medial pontine nuclei (Brodal, 1979; Schmahmann, 1996; Schmahmann and Pandya, 1997a,b), whereas parietal regions project to the lateral pontine nuclei (Brodal, 1979, 1982; Glickstein et al., 1994). However, these pathways have yet to be definitively mapped *in vivo* in human studies.

Several tractography studies in humans have identified cerebro-cerebellar white matter tracts *in vivo* (e.g., Habas and Cabanis, 2007a,b; Doron et al., 2010; Anderson et al., 2011; Palesi et al., 2014). Most of those studies used a diffusion tensor imaging model for tracking cerebro-cerebellar connections. The tensor model is unable to resolve multiple fiber orientations within one voxel (Mori and van Zijl, 2002); hence it cannot resolve crossing fibers. Therefore, studies that used tensor models were only able to reconstruct ipsilateral pathways from cerebellar peduncles to cerebral cortex (Salamon et al., 2007; Habas and Cabanis, 2007a,b; Jissendi et al., 2008; Doron et al., 2010; Anderson et al., 2011) but were unable to reconstruct crossing cerebellar fibers. Using advanced diffusion MRI tractography, Palesi et al. (2014) were able to track in the human brain the crossed cerebro-thalamo-cerebellar pathways, the majority of which were found to connect the cerebellum and frontal, prefrontal and temporal cortices; only very few streamlines were reconstructed between the cerebellum and the parietal cortex.

Frontal and prefrontal white matter connections with the cerebellum have been demonstrated by several studies (e.g., Doron et al., 2010; Palesi et al., 2014), but only one study by Doron et al. (2010) examined the connection between subdivisions of the frontal cortex and cerebellar peduncles. Doron and co-workers found that the majority of the cortico-pontine projections connecting frontal regions with the cerebellum are between the precentral gyrus, middle/superior frontal gyrus and cerebellar peduncle. The connectivity identified from the IFG was only sparse. Based on this it was concluded that the observed co-activations between the IFG and the superior cerebellum in functional neuroimaging studies of verbal working memory (Chen and Desmond, 2005a,b) may be part of a functional network that lacks direct anatomical connection.

It is within the context of inconclusive findings on white matter connections between the IFG and the cerebellum as well as between the inferior parietal gyrus and the cerebellum (Glickstein, 2000; Clower et al., 2005; Doron et al., 2010; Palesi et al., 2014), the current study was conducted to examine the anatomical connectivity between these regions. We aimed to trace the following white matter pathways: (1) For the fronto-ponto-cerebellar network, the tract descending from the left inferior frontal gyrus (IFG) to the pons and the tract from the pons to the right superior cerebellar lobule VI/crusI (sCERE); (2) For the parieto-ponto-cerebellar network, the tract between the left inferior parietal lobule (IPL) and the pons and the tract from the pons to the right inferior cerebellar lobule VIIb/VIIIa (iCERE) and (3) for the cerebello-thalamo-frontal pathway, ascending tracts from the right sCERE/iCERE via the thalamus to the left IFG. Additionally, we traced the tract between left IFG and left IPL as frontal and parietal regions are known to be essential to the verbal working memory (for review see Eriksson et al., 2015) and the tracts on the other side of the brain that correspond to the tracts of interest listed above. The tracts were traced by using anatomically guided deterministic tractography with a fiber tracking algorithm with quantitative anisotropy. This means that the tracts trajectories/orientations were estimated and reconstructed by using quantitative anisotropy algorithm which has been shown to have better spatial resolution than fractional anisotropy algorithm as it is less sensitive to partial volume effect (Yeh et al., 2013).

Anatomical connectivity imposes constrains on effective connectivity but it does not determine it because presence of structural synaptic connection is not enough to determine if that synaptic connection will engage in particular process or not (Stephan et al., 2009b). Hence, providing evidence for the existence of structural connectivity only between cerebro-cerebellar regions contributing to verbal working memory is not enough to establish whether these particular white matter connections are engaged in verbal working memory processes or not. Therefore, once the connectivity between fronto-ponto-cerebel, parieto-ponto-cerebellar and cerebello-thalamo-frontal networks were reconstructed, we carried out further investigations as follows: First, we wanted to examine if the microstructure of these white matter tracts contribute to individual differences in verbal working memory performance, especially given that previous brain stimulation and functional neuroimaging studies have shown that there is a relationship between activation of cerebellar regions and task performance (e.g., Desmond et al., 2005; Marvel and Desmond, 2010a). More specifically, previous functional neuroimaging studies linked the left IFG and right superior cerebellum with the articulatory rehearsal and left IPL and right inferior cerebellum with the phonological storage (Chen and Desmond, 2005b; Ng et al., 2016). Additionally, stimulation studies demonstrated that the increased activity in the inferior cerebellum is associated with increased item recognition capacity (Macher et al., 2014) and the transient lesion to the superior cerebellum is associated with increased response time during Sternberg memory task (Desmond et al., 2005). Considering these findings, we hypothesized that greater white matter integrity of the tracts connecting IFG via pons to the superior cerebellum will be associated with faster response times in verbal working memory, whereas greater white matter integrity of the tracts connecting IPL via pons to the inferior cerebellum will be associated with higher accuracy rates. Second, we examined whether the white matter tracts included in the cerebro-cerebellar network underlying verbal working memory are related to the dynamics of the interactions between cerebro-cerebellar regions implicated in verbal working memory. Given that the verbal stimuli was presented to participants visually, DCM models included not only nodes that support verbal working memory processing but also regions important for processing of visual stimuli – the inferior occipital gyrus (IOG) and fusiform gyrus (FG). For the visual input and analysis, the model postulated that activity in the IOG—involved for primary visual analysis—was modulated by visual stimulation at the driving input region. Intrinsic connectivity between the IOG and the FG, which has a pertinent role in secondary visual analysis, was also proposed in the present model based on the previous findings from Wu et al. (2014). It is important to note that even though virus tracing studies have indicated that projections from frontal regions enter to medial portions of the pons and then to the superior cerebellum whereas projections from parietal regions enter more lateral parts of the pons, the functional significance of the medial and lateral pons for working memory function in human functional neuroimaging studies has yet to be established. As the spatial resolution for the functional data to have two distinct activations in the pons and to show this separation of medial from lateral pontine nuclei is limited, we wanted to use a more parsimonious model without the pons to conduct the effective functional study. Interestingly, in the initial stages of white matter pathways reconstruction, we found that the tractography failed to be established without including the pons. Thus, given the limited spatial resolution we reconstructed white matter pathways by adding pons as a broad point for tracking of cerebro-cerebellar projections without separating into two nodes of lateral and medial pontine nuclei. Despite this, the tractography was successful and the pons was found to be a crucial node for the reconstruction of white matter pathways. Therefore, we reasoned, that pons may be a significant node the DCM models of working memory we created as well. To test this, we had models with and without the pons as a broad node (rather than more specific lateral and medial pons nodes). In order to do this, we investigated if Bayesian model selection (BMS) of dynamic causal models (DCM) informed by the tractography results (models with pons) would have a higher degree of confidence in model inference for the models including pons (informed) than for the models without pons (uninformed models). Following this, we examined if structural connectivity can predict effective connectivity within this network.

## Materials and Methods

### Participants

Thirty-five young adults (17 females, 18 males; mean age = 22.86; *SD* = 1.90) participated in DCM study but only 31 participants took part in our DSI study as for some of study participants we were unable to collect DSI data due to technical difficulties. All participants were right-handed (mean Laterality Quotient = 96.67; *SD* = 6.37 on Edinburgh Handedness Inventory; Oldfield, 1971) and had normal or corrected to normal vision, and they reported no history of neurological or mental illness. The study was approved by the Institutional Review Board at Nanyang Technological University and the National Health Group in Singapore. Written informed consent was obtained from all participants.

### Task Description

**FIGURE 1:**
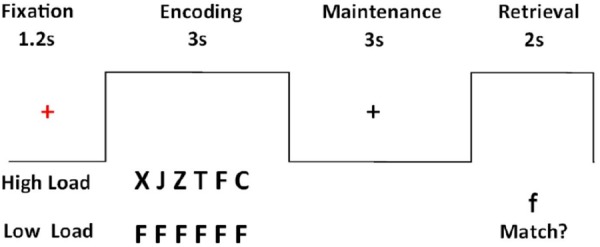
Sternberg’s verbal working memory task.

Participants performed a modified version of a Sternberg verbal working memory task (Sternberg, 1966) within a block design (Figure 1). The task was presented in alternating blocks of high-load and low-load trials adding up to a total of 20 high-load and 20 low-load trials. The presentation of each trial started with a red fixation cross in the middle of the screen which appeared for 1.2 s. Then a horizontal array of six different (high load) or identical (low load) letters was presented for 3 s (encoding phase). While watching the letters, participants were instructed to rehearse them subvocally and to continue doing so during the maintenance phase when the letters were no longer displayed on the screen. The maintenance phase lasted 3 s and afterward a probe letter was presented for 2 s. The participants’ task was to decide if the probe letter matched one of the previously displayed letters and respond accordingly by pressing a button. The task was presented using E-Prime 2.0 software (Psychology Software Tools, Inc, Pittsburgh, PA, United States) in the scanner. Prior to the scanning procedure, all participants had a practice session outside the scanner to become familiar with the task. The task had two runs and the order of run presentation was counterbalanced across participants.

### MRI Data Acquisition

All neuroimaging data were acquired on a 3T scanner (Tim Trio, Siemens, Erlangen, Germany) with a 32-channel quadrature head coil. To obtain an anatomical reference, high-resolution T1-weighted imaging was performed using a 3D magnetization-prepared rapid gradient echo sequence and the following parameters: repetition time (TR) = 2300 ms, echo time (TE) = 1.9 ms, flip angle = 9°, field of view (FOV) = 256 mm × 256 mm × 192 mm, acquisition matrix = 256 mm × 256 mm × 192 mm resulting in isotropic spatial resolution of 1 mm^3^. Diffusion data were acquired using a twice-refocused balanced echo spin-echo diffusion EPI sequence to minimize eddy-current induced image distortions (Reese et al., 2003). The following image acquisition parameters were used: TR = 7200 ms, TE = 136 ms, slice thickness = 3 mm, 45 slices with no gap, interleaved, FOV = 220 mm x 220 mm, acquisition matrix = 74 × 74, giving an in-plane voxel size of 3 mm × 3 mm. Here, we adopted diffusion spectrum imaging (DSI) as our data acquisition scheme owing to its ability to resolve crossing fibers (Wedeen et al., 2005). To reduce the scan time, the diffusion weighting was distributed along a grid of 128 directions filled in the half sphere of the diffusion-encoding space (q-space) with a maximum diffusion sensitivity of 7000 s/mm^2^.

Functional MRI for verbal working memory was performed using a gradient-echo EPI sequence with the following parameters: TR = 2500 ms, TE = 29 ms, 48 interleaved slices, FOV = 225 mm, slice thickness = 3.5 mm, acquisition matrix = 64 × 64, giving an in-plane voxel size of 3.5 × 3.5 mm^2^. A total of 2 runs, 160 measurements per run, were administered. The total scan time, including T1-weighted images, DSI and functional MRI, was approximately 36 min.

### Data Analysis

#### Diffusion Spectrum Imaging

All DSI analysis, including reconstruction of the probability density function (PDF), orientation distribution function (ODF), tractography and computation of generalized fractional anisotropy (GFA) values were conducted using DSI Studio^1^. The acquired half-sphere data were projected to fill the other half of the sphere based on the fact that data in the q-space are real and symmetrical about the origin. The grid points in the eight corners outside the sphere were filled with zeros. 3D Fourier transform was performed to obtain the PDF (Callaghan et al., 1991), and the ODF in 362 radial directions (sixfold tessellated icosahedron) was obtained by computing the second moment of PDF along each corresponding direction (Wedeen et al., 2005). GFA is a DSI index equivalent to the fractional anisotropy (FA) in diffusion tensor imaging (DTI) (Gorczewski et al., 2009; Fritzsche et al., 2010). The GFA index was computed for each voxel by the following formula: (standard deviation of the ODF)/(root mean square of the ODF) (Tuch, 2004). The GFA value varies from 0 (being completely isotropic) to 1 (being completely anisotropic) and has been shown to reflect microscopic property related to myelination, axonal density, axonal diameter or directional coherence of the white matter (Gorczewski et al., 2009; Fritzsche et al., 2010).

The primary 3 loops (fronto-ponto cerebellar, parieto-ponto-cerebellar, and cerebello-thalamo-frontal pathways)that were examined in the current study were derived based on findings of animal studies that have provided evidence that the cerebro-cerebellar white matter system includes contralateral cortico-ponto-cerebellar pathways (CPC) (Brodal, 1979; Schmahmann, 1996) that project from cortical regions through the pons to the cerebellum and contralateral cerebello-thalamo-cortical pathways (CTC) that connect the cerebellum through the thalamus and red nucleus with the cerebral cortex (Middleton and Strick, 1994, 2001; Schmahmann, 1996; Kelly and Strick, 2003). This was further supported by diffusion studies that reconstructed in the human brain the crossed cerebro-thalamo-cerebellar pathways (Palesi et al., 2014) and demonstrated that there are frontal and prefrontal white matter connections with the cerebellum (e.g., Doron et al., 2010; Palesi et al., 2014). A template-based approach (Chen et al., 2015) was employed to reconstruct white matter tracts for the cerebro-cerebellar verbal working memory network that included fronto-ponto-cerebellar, parieto-ponto-cerebellar, and cerebello-thalamo-frontal loops. Regions of interests (ROIs) that were used for tractography included: the left IFG, left IPL, pons, left thalamus, right sCERE (lobule VI/crusI) and right iCERE (lobule VIIb/VIIIa). The non-cerebellar ROIs were selected using an automated anatomical labeling (AAL) system (Tzourio-Mazoyer et al., 2002), whereas cerebellar ROIs were selected using the cerebellum atlas implemented in SUIT toolbox (Diedrichsen, 2006). Targeted fiber tracts were reconstructed on the NTU-122 DSI template (Hsu et al., 2015) using a streamline-based algorithm that was adapted for DSI data (Yeh and Tseng, 2013). Voxels with GFA values higher than the given threshold of 0.03 were selected as the white matter regions and were later used as seed voxels for tractography over the whole brain. The GFA threshold value of 0.03 for white matter masking was comparable with the FA value of 0.05 used by Berman et al. based on HARDI data (Berman et al., 2008). For each seed voxel, the proceeding orientation for the next step was decided by the angular deviation between the primary orientation within the seed voxel and all the fiber orientations of its nearest voxels; the most coincident orientation with the minimum angular deviation was chosen. For the targeted tracts in this study, the angle threshold was 60°. By moving the seed point with a proceeding length of 0.5 voxel for each step along the most coincident orientation, the new starting point was then obtained. We restricted the range of tract length as 50–100 mm to ensure the accuracy of the segmented tracts and the fiber number was set to include minimum 300 fibers for each targeted tract bundle. Once the angle deviations were higher than a given angular threshold, the tracking would stop. These procedures were repeated, and the targeted fiber tracts were then obtained. Once the tract bundle was reconstructed, the entire trajectory was verified to ensure its consistency with the established anatomical landmarks. Finally, the eight targeted tracts included in the verbal working memory network. For fronto-ponto-cerebellar pathway the reconstructed tracts included the fronto-pontine tract descending from the left IFG to the pons, and the ponto-cerebellar tract connecting the pons to right sCERE. For the parieto-ponto-cerebellar pathway the reconstructed tracts included the parieto-pontine tract descending from the left IPL to the pons and the ponto-cerebellar tract connecting the pons to right iCERE. The tracts that were reconstructed for the cerebello-thalamo-frontal pathway included tracts ascending from the right sCERE and right iCERE to left thalamus and tract from the left thalamus to left IFG. In this study, we also segmented the cortical tracts between the IFG to the IPL in both hemispheres. Additionally, the corresponding contralateral tracts to the tracts of interest we traced. These included (1) tract descending from the right IFG to the pons, tract connecting the pons to left sCERE, (2) tract descending from the right IPL to the pons and tract connecting the pons left iCERE, (3) tracts ascending from the left sCERE and left iCERE to left thalamus and tract from the right thalamus to right IFG. A study-specific DSI template (SSDT) was constructed from all the recruited participants using a spatial registration method within the framework of Large Deformation Diffeomorphic Metric Mapping (LDDMM) (Hsu et al., 2012). The registration strategy included both the gray matter anatomy provided by T1-weighted images and the white matter fiber structures provided by DSI images. The SSDT was then registered to the NTU-122 DSI template by the same registration strategy. The sampling coordinates of the targeted tracts were then transformed from the NTU-122 DSI template to the SSDT and from the SSDT to individual DSI datasets according to established transformations (Chen et al., 2015). Finally, the GFA values of each targeted tract were sampled on the corresponding sampling coordinates in the native DSI space. The tract integrity for each targeted tract was determined by averaging the GFA values of each tract bundle.

#### Dynamic Causal Modeling

DCM12 implemented in spm12 (v6470) was used for effective connectivity analysis. DCM for fMRI uses coupled differential equations in which the interactions between neural states can be (1) endogenous or intrinsic (the interregional influences that occur in the absence of modulating experimental effects); (2) modulatory connections (the changes in the intrinsic connectivity between regions that are induced by the conditions of the experimental design and (3) driving inputs (the changes in regional activity induced by the direct influence of stimuli) (Friston et al., 2003; Stephan et al., 2007). Before conducting DCM analysis, images were preprocessed using a conventional protocol of slice timing correction, realignment, co-registration, normalization and smoothing in SPM12 (Wellcome Trust Centre for Neuroimaging, London, United Kingdom^2^). Slice timing correction was aligned to the middle slice of the EPI images to correct for temporal lags in image acquisition followed by the realignment of images to the first volume. Structural images were then co-registered to the EPI images before they are normalized to the Montreal Neurological Institute (MNI) space. Lastly, images were smoothed using a Gaussian kernel of 8 mm full-width at half-maximum (FWHM). A general linear model was used to examine task-related individual activations. The created design matrix included two conditions – high memory load and low memory load and also six motion parameters. The correct and incorrect trials were modeled at the first level of analysis. A high load > low load contrast was produced for each subject. Then a group level analysis was conducted using a random effects model (Friston et al., 1999). The statistical threshold was set to *p* < 0.001 uncorrected with minimum cluster size of 20 voxels.

Volumes of interest (VOIs) in each of the models included the following *a priori* chosen VOIs: IFG, IPL, thalamus, sCERE, iCERE, IOG, FG. The first 5 VOIs included in the model were selected based on past fMRI findings that demonstrated the importance of these regions for verbal working memory and the remaining 2 VOIs were included for their importance in the analysis of visually presented stimuli (Chen and Desmond, 2005b; Marvel and Desmond, 2010a; Stoodley et al., 2012; E et al., 2014; Wu et al., 2014).

**FIGURE 2:**
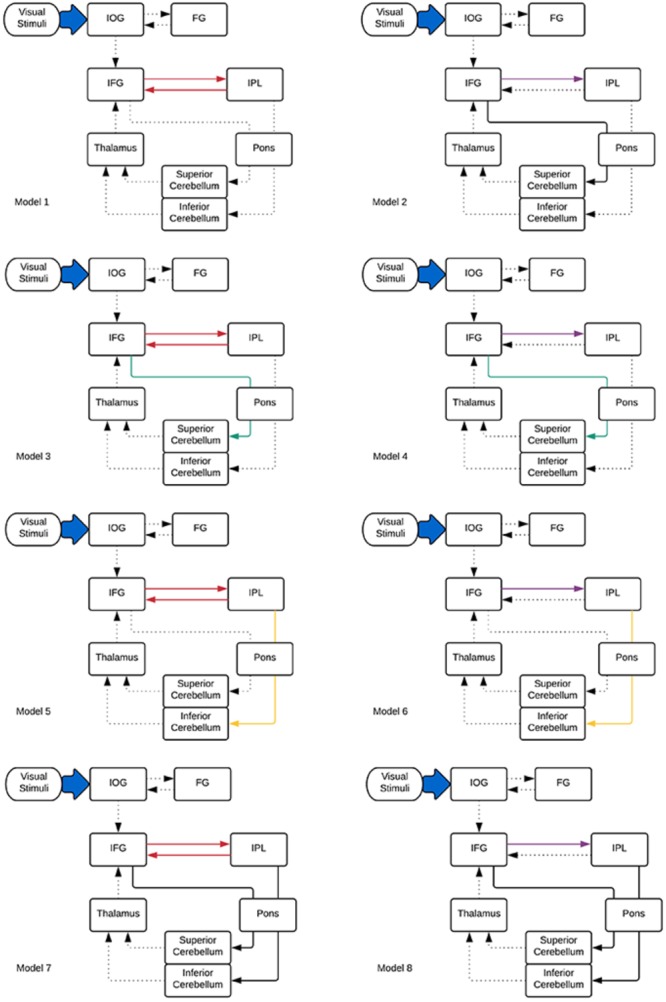
DCM models informed by the tractography results specified for effective connectivity analysis. Dotted lines indicate intrinsic effects, red, green, yellow, and purple lines represent the modulatory effects. Driving input is indicated by the blue arrow.

Considering these ROIs, eight possible pathways were created as shown in Figure 2. Model 1 and model 2 were proposed based on the phonological loop (Baddeley, 2003)—the models with bilateral modulatory effect from the IFG to the IPL and the IPL back to the IFG (odd-numbered models; modulatory connectivity denoted by bolded red lines) and models with only modulatory effects from the IFG to the IPL (even-numbered models; modulatory connections denoted by bolded purple lines). The next six models were postulated based on the two cerebro-cerebellar networks established by Chen and Desmond (2005b). Here, we considered the modulatory effects of verbal working memory task load on either the frontal/ superior cerebellar articulatory control system (Model 3 and 4; denoted by bolded green lines) or the parietal/ inferior cerebellar phonological storage system (Model 5 and 6; denoted by bolded yellow lines). Lastly, two more models (model 7 and 8; denoted by bolded black lines) with the combination of modulatory effect on the two cerebro-cerebellar pathways were considered. This resulted in a total of eight models for comparison. Pons were not included in our initial modeling. However, the VOI in the pons was added to the DCM models to match the anatomical white matter pathways nodes reconstructed in this study (Figure 2). Thus, we had two sets of models, with and without the pons. The anatomical masks for all VOIs were then applied to the activation map to obtain the group maxima for each of the individual VOI. These were selected based on the largest *T*-value within VOI for one-sample t-test on all participants in the group (*p* < 0.001 uncorrected with minimum ≥ 20 voxels). The peak coordinates of the VOIs were as follows: FG (*x* = -39, *y* = -66, *z* = -12), IOG (*x* = -39, *y* = 78, *z* = -9), IFG (*x* = -42, *y* = 3, *z* = 27), IPL (*x* = -27, *y* = -60, *z* = 42), thalamus (*x* = -15, *y* = -9, *z* = -6), sCERE (*x* = 21, *y* = -60, *z* = -27), iCERE (*x* = 21, *y* = -72, *z* = -51), pons (*x* = -6, *y* = -42, *z* = -42). At the individual subject level, the activation maps were thresholded at *p* < 0.5 (uncorrected) and the ROI masks were applied. The VOIs were constrained to be located within the boundaries of ROIs defined by anatomical masks created for those regions using Anatomical Automatic Labeling (AAL) (Tzourio-Mazoyer et al., 2002) within the WFU Pick Atlas tool (Maldjian et al., 2003). VOIs were created using 8mm radius spheres centered at the peak coordinates. The eigenvariates of the activated voxels within the VOIs were extracted as regional responses. Model comparison was performed using random effects BMS in DCM12. The protected exceedance probabilities (Stephan et al., 2009a; Rigoux et al., 2014) were computed at the group level in order to identify the most optimal model for our dataset. Then the random effects Bayesian model averaging (BMA) was used to obtain average connectivity estimates.

#### Correlational Analysis

Pearson correlational analysis was used to examine (1) the relationship between structural and effective connectivity and (2) the relationship between structural connectivity and verbal working memory task performance. To investigate the relationship between structural and effective connectivity, examined were correlations between the values of modulatory parameters extracted from the DCM.Ep.B matrix of model 8 for each participant and mean GFA values in the matching white matter tracts (i.e., those that displayed modulatory effect in model 8). In order to asses if there is relationship between task performance and structural connectivity, correlational analysis was performed for the mean GFA values of cerebro-cerebellar tracts and verbal working memory task performance (mean response time and mean accuracy rates for high load only). All behavioral analyses were performed using SPSS23 (SPSS Inc., United States).

## Results

### Behavioral Results

Paired-samples *t*-tests were used to evaluate high versus low load differences in accuracy and response times that were measured in percentages and milliseconds respectively. This revealed that mean accuracy for low load (*M* = 0.99, *SD* = 0.02) was significantly higher [*t*(30) = -4.64, *p* < 0.0001] than mean accuracy for high load (*M* = 0.95, *SD* = 0.04). Mean response time for high load (*M* = 930.41, *SD* = 169.78) was significantly longer [*t*(30) = 15.52, *p* < 0.0001] than mean response time for low load (*M* = 645.01, *SD* = 111.95).

### Tractography Results

**FIGURE 3:**
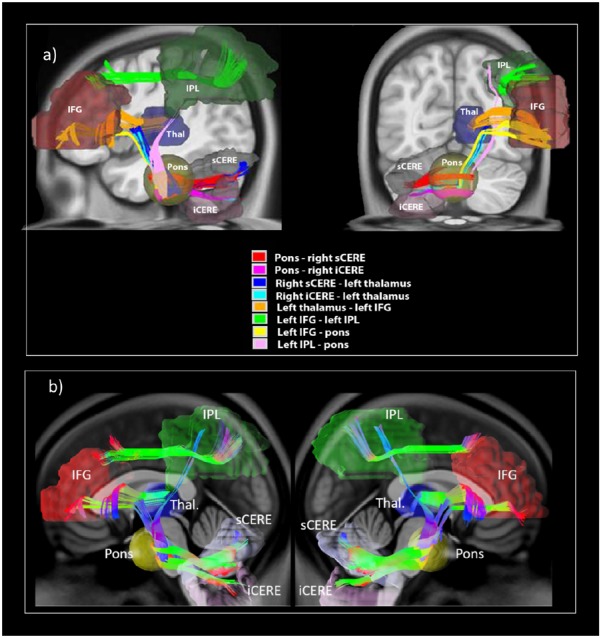
White matter tracts reconstructed in the current study illustrating **(a)** reconstructed white matter tracts; **(b)** shows reconstructed left cerebro-right cerebellar tracts (on the left) and reconstructed right cerebro-left cerebellar tracts (on the right).

All eight tracts from the verbal working memory network, namely the left IFG to pons, pons to right sCERE, left IPL to pons, pons to right iCERE, left IFG to left IPL, right iCERE and right sCERE to thalamus, and thalamus to left IFG and corresponding contralateral tracts were successfully reconstructed. Figure 3 shows group-average image of the reconstructed tracts of interests and additionally IFG-IPL tract. Means and standard deviations of group GFA values for all the reconstructed tracts are shown in Table 1.

**Table 1:** Mean GFA values and standard deviations for cerebro- cerebellar working memory network.

Cerebro-cerebellar white matter tracts (*N* = 31)	GFA value	
	Mean	*SD*
Left Inferior Frontal Gyrus – Left Inferior Parietal Lobule	0.32	0.02
Pons – Left Inferior Frontal Gyrus	0.25	0.01
Pons – Left Inferior Parietal Lobule	0.35	0.01
Pons – Right Superior Cerebellum	0.25	0.02
Pons – Right Inferior Cerebellum	0.36	0.02
Right Superior Cerebellum – Left Thalamus	0.28	0.01
Right Inferior Cerebellum – Left Thalamus	0.32	0.01
Thalamus – Left Inferior Frontal Gyrus	0.29	0.02
Right Inferior Frontal Gyrus – Right Inferior Parietal Lobule	0.29	0.03
Pons – Right Inferior Frontal Gyrus	0.24	0.01
Pons – Right Inferior Parietal Lobule	0.34	0.01
Pons – Left Superior Cerebellum	0.26	0.01
Pons – Left Inferior Cerebellum	0.36	0.02
Left Superior Cerebellum – Right Thalamus	0.30	0.01
Left Inferior Cerebellum – Right Thalamus	0.33	0.01
Thalamus – Right Inferior Frontal Gyrus	0.28	0.01

### Structural Cerebro-Cerebellar Connectivity Predicts Verbal Working Memory Task Performance

The tracts of interest in the left cerebral-right cerebellar network included: (1) IPL-pons, pons-iCERE and iCERE-thalamus which were used for correlational analysis with accuracy rates and (2) IFG-pons, pons-sCERE and sCERE-thalamus which were used for correlational analysis with the response times. Response time was positively correlated with the mean GFA value of pons-sCERE tract (*r* = 0.43, *p* = 0.013), but not with the IFG-pons tract or the sCERE-thalamus tract. The identified correlation was still significant when corrected for multiple comparisons (*p* < 0.016) were accounted for with Bonferroni correction. Task Accuracy was not significantly correlated with the mean GFA of any tracts from the parietal-inferior cerebellum loop (*p* > 0.05).

For the corresponding white matter tracts on the contralateral side of the brain to the tracts reconstructed in the current study, no significant correlations were found between mean GFA values of the tracts from the right IPL – left iCERE loop and accuracy rates. For the tracts from the right IFG – left sCERE loop the significant correlation that was found between response time and mean GFA values of the pons and left superior cerebellum (*r* = 0.55, *p* = 0.001), which is analogous to findings for left cerebral – right cerebellar white matter pathways from verbal working memory network.

### FMRI Analysis

**FIGURE 4:**
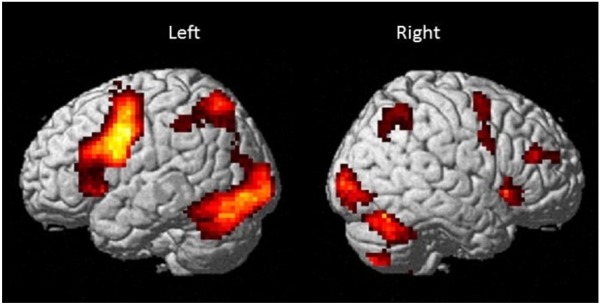
Brain activation map for the Sternberg verbal working memory task (High Load > Low Load; *p* < 0.001 uncorrected with minimum cluster ≥ 20).

Results are shown in Table 2 and Figure 4 and indicate strong left cerebral, right cerebellar dominance. This is in line with past research examining the underlying neural network during verbal working memory tasks that found similar pattern of activation in the left IFG, left IPL, right inferior cerebellum and right superior cerebellum (Baddeley, 2003; Chein et al., 2003; Chen and Desmond, 2005a).

**Table 2:** Montreal Neurological Institute (MNI) peak coordinates within the significant clusters for the effects of load (High load > Low load).

Region	Left/Right	Cluster Size (voxel)	MNI Coordinates			*T*
			*x*	*y*	*z*	
Precentral Gyrus	Left	882	–51	3	45	13.00
Precentral Gyrus	Left		–45	3	30	12.09
Insula	Left		–33	24	3	9.21
Superior Cerebellum (Lobule VI)	Right	2808	21	–60	–27	12.87
Superior Cerebellum (Lobule VI)	Right		12	–72	–21	10.97
Medial Occipital Region	Left		–5	–90	0	10.84
Superior Parietal Region	Left	383	–24	–60	42	11.58
Middle Occipital Region	Left		–27	–69	27	8.71
Medial Frontal Region	Left	350	–3	15	45	10.18
Medial Frontal Region	Left		–6	6	57	9.21
Limbic Region	Right		9	21	36	8.79
	Left	608	–21	–6	12	9.56
Caudate Nucleus	Right		15	0	18	8.83
	Right		12	–3	0	8.05

*Thresholded at FWE < 0.05 (corrected).*

### DCM Results

**FIGURE 5:**
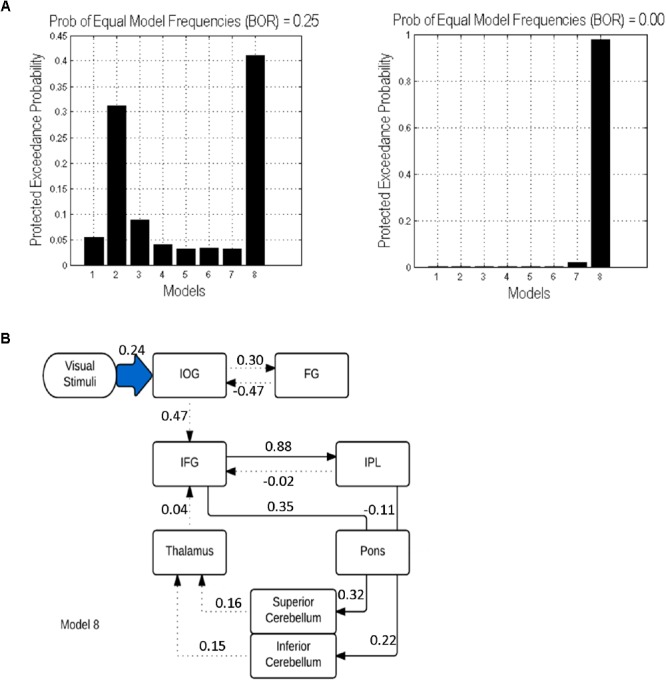
DCM results: **(A)** Protected exceedance probability for 8 specified models uninformed (left) and informed (right) by tractography results. **(B)** The optimal model 8 informed by tractography results. Dotted lines indicate intrinsic effects, continuous lines represent the modulatory effects. Driving input is indicated by the blue shaded arrow.

In both cases (with and without the pons), model 8 was found to be the best fitting model. However, adding the pons to the model resulted in an increase of the protected exceedance probability from 40 to 98% (Figure 5). In this model, the forward modulated connections were between: (1) IFG and IPL, (2) IFG and pons, (3) pons and sCERE, (4) IPL and pons and (5) pons and iCERE. All modulatory connections were excitatory apart from the connection from IPL to pons which was inhibitory. Connectivity values as estimated by random effects BMA are shown in Figure 5.

### Relationship Between Anatomical and Effective Connectivity

We investigated potential correlations between the values of modulatory parameters extracted from the DCM.Ep.B matrix of model 8 for each participant and mean GFA values in the matching white matter tracts (i.e., those that displayed modulatory effect in model 8). However, we did not find any significant correlations between structural and modulatory connectivity (*p* > 0.05), which suggests that there is no direct relationship between effective and structural connectivity within our verbal working memory network.

## Discussion

In the current study, DSI was used to conduct deterministic tractography of fronto-cerebellar and parieto-cerebellar white matter tracts connecting cerebro-cerebellar regions involved in verbal working memory. Once the tracts were reconstructed, we examined whether the microstructure of these tracts was associated with verbal working memory performance. We also tested the relationship between structural and effective connectivity in two ways: We first examined whether providing DCM models with more precise information about the underlying white matter structure between regions included in the models would improve the robustness of model selection. Then we investigated whether the structural connectivity could predict effective connectivity in the most optimal model.

There are three main findings of the current study. (1) In the study, we demonstrated *in vivo* structural tract connectivity between the left IFG via pons to the right sCERE, and between the left IPL and right iCERE via the pons using DSI tractography. (2) Our findings showed that creating DCM models that more closely resemble underlying structural connectivity resulted in a higher degree of BMS confidence. (3) The findings of the current study show that greater structural connectivity of the pons-superior cerebellum tract is associated with slower responses for verbal working memory task. This is consistent with the finding demonstrating importance of superior cerebellum for verbal working memory (Desmond et al., 2005). Past studies investigating relationship between white matter microstructure and memory have often focused on cortico-cortical pathways (e.g., Takeuchi et al., 2010; Charlton et al., 2013), even though the relationship between white matter of cerebellar peduncles and cognitive functions such as memory (Takahashi et al., 2010) or reading skills (Travis et al., 2015) has been established.

The present study, however, found that the GFA of pons-superior cerebellum tract is associated with slower responses for the verbal working memory task. A possible mechanism explaining this relationship is that greater connectivity of this tract (expressed by the GFA index) facilitates quicker and higher fidelity neural transmission between the pons and superior cerebellum. This explanation would be supported by the fact that myelin thickness in the myelinated neuron fibers (Waxman, 1980) and axonal diameter are important determinants of neuronal conduction velocity (Arbuthnott et al., 1980). The GFA value has been shown to reflect the degree in myelination, axonal diameter and membrane density, and directional coherence of white matter (Gorczewski et al., 2009; Fritzsche et al., 2010). The facilitation of quicker and higher fidelity neural transmission between the pons and the superior cerebellum may lead to more comparison and updating processes in the cerebellum as it is likely that more information about the rehearsed representation is received. The increased rehearsal processing would thus lead to slower response times. If this explanation was true, rehearsal processing providing more detailed information about rehearsed items should be associated with higher accuracy rates. This was not observed in this study, which may be due to the fact that variation in accuracy rates was small. Further studies with more difficult verbal working memory tasks could be used to test this explanation. Although the suggested explanation for the identified inverse relationship between white matter integrity of the pons-superior cerebellum and response time is speculative, it is indirectly supported by the findings of Yamamoto et al. (2015) who reported that small response conflict is associated with finer structural connectivity when compared to participants with large response conflict.

In the current study, we failed to find any relationship between effective and structural connectivity for verbal working memory as hypothesized. There was no significant relationship between GFA values of cerebro-cerebellar white matter tracts and modulatory connectivity for those tracts. The lack of direct relationship between the structural and effective connectivity may result from the fact that changes in effective connectivity may be additionally influenced by neural oscillations and the connectivity between areas may be direct or indirect (Furl, 2015). However, the findings showed that BMS of DCM models informed by the tractography results has a higher degree of confidence in model inference for the informed models compared to the uninformed models; however, we did not find that structural connectivity predicts effective connectivity. Thus, tractography results can be useful in improving inference about effective connectivity, even when there is no direct relationship between effective and structural connectivity. This is consistent with previous research showing that information about anatomical connectivity is beneficial in modeling functional dynamics between regions but it does not predict it (Stephan et al., 2009b). In our study, the intermediate ROI in the pons was needed to reconstruct tracts implicated in verbal working memory (IFG-sCERE and IPL-iCERE), even though the functional relevance of the pons for verbal working memory was not detected in fMRI studies (e.g., Chen and Desmond, 2005a; Marvel and Desmond, 2010a; Macher et al., 2014). This suggests that tractography could be employed more widely in improving DCM models by informing researchers about brain regions that are needed as midpoints to reconstruct white matter pathways between functionally co-activated areas. Additionally, our findings demonstrate that verbal working memory-related changes in effective connectivity occur not only for fronto-parietal regions but for fronto-ponto-cerebellar and parieto-ponto-cerebellar regions. More specifically, in the current study we found positive connectivity between IFG and pons and sCERE. This suggests that activity from IFG enhances activity in pons and subsequently in superior cerebellum. Past functional neuroimaging findings linked co-activated fronto-cerebellar regions during verbal working memory task with articulatory control (e.g., Chen and Desmond, 2005a). Therefore, the excitatory connectivity from IFG and pons on superior cerebellum is also likely to support articulatory control during verbal working memory and the superior cerebellum could enhance this function by providing automation and predictive mechanisms (Caligiore et al., 2016). For the parieto-ponto-cerebellar connectivity, we found that IPL exerts inhibitory effects over the pons and pons have excitatory influence over the inferior cerebellum. A possible explanation of this finding may be that the inhibitory influence from parietal cortex over pons may help to sustain activated verbal representations free of any additional interference.

The present study has a number of limitations. First, the DSI tractography does not permit afferent and efferent tracts to be distinguished, and therefore the direction of the information flow within the identified cerebro-cerebellar white matter tracts cannot be determined (Thomas et al., 2014; Daducci et al., 2016). Second, our attempts to establish this relationship between Task Accuracy and cerebellar tracts could have been hindered by the fact that the diffusion signal near the inferior cerebellar regions is characterized by more noise than in other parts of the brain. In addition, participants had high accuracy scores with little within-group variation, thus a possible relationship between verbal working memory accuracy and white matter connectivity of parieto-ponto-cerebellar network cannot be excluded. Future studies could help to determine whether such a relationship exists. Additionally, utilizing an event-related design may also help to determine if relationship between verbal working memory accuracy and white matter connectivity of parieto-ponto-cerebellar network exists with the use of the additional measure provided by the percent MRI signal change for high and low loads.

## Conclusion

The current study is the first to demonstrated *in vivo* structural tract connectivity of the left IFG via the pons to the right sCERE, and the left IPL via the pons with the right iCERE using DSI tractography. We found that structural connectivity of the reconstructed cerebro-cerebellar networks relates functionally to verbal working memory in two ways: First, constructed DCM models that resembled the underlying anatomical pathways more closely had a higher degree of confidence in BMS. Second, it was found that the increasing connectivity of the ponto- superior cerebellar tract was correlated with increased response time for the verbal working memory task, implying that the structure of ponto-cerebellar projection contributes to individual differences in verbal working memory. These findings have implications for understanding the variability in verbal working memory performance by considering the inclusion of the functional contribution of cerebellar white matter pathways. Future research in clinical populations can further help to determine how cerebellar white matter microstructure contributes to intact and deficient verbal working memory performance.

## Author Contributions

MS-E conducted the fMRI/DCM analyses, contributed to the DSI analysis, and wrote the manuscript. Y-CL conducted the DSI analysis and wrote the methods for this process. Y-CH and Y-JC contributed to the DSI analyses. FYK contributed to the DCM analyses and related methods write-up. K-HC contributed to setting up the EPI and DSI pulses sequences for data acquisition. W-YT provided the overall DSI analysis conceptualization and contributed to the paper conceptualization write-up. SHAC provided overall conceptualization of the study, oversaw study design, implementation, data collection, analysis and write-up of the manuscript. SHAC is also the principle investigator of the grant that supported this study.

## Conflict of Interest Statement

The authors declare that the research was conducted in the absence of any commercial or financial relationships that could be construed as a potential conflict of interest.

## Footnotes

1 http://dsi-studio.labsolver.org

2 http://www.fil.ion.ucl.ac.uk/spm/
